# Assessment of osteopontin in early breast cancer: correlative study in a randomised clinical trial

**DOI:** 10.1186/bcr3600

**Published:** 2014-01-22

**Authors:** Vivien HC Bramwell, Alan B Tuck, Judith-Anne W Chapman, Pieter H Anborgh, Carl O Postenka, Waleed Al-Katib, Lois E Shepherd, Lei Han, Carolyn F Wilson, Kathleen I Pritchard, Michael N Pollak, Ann F Chambers

**Affiliations:** 1 Tom Baker Cancer Centre, University of Calgary, Calgary, AB T2N 4N2, Canada; 2 London Regional Cancer Program, University of Western Ontario, 790 Commissioners Road East, London, ON N6A 4 L6, Canada; 3 NCIC Clinical Trials Group, Queen’s University, Kingston, ON K7L 3N6, Canada; 4 Sunnybrook Odette Regional Cancer Centre, University of Toronto, Toronto, ON M4N 3M5, Canada; 5 Jewish General Hospital, McGill University, Montreal, QC H3T 1E2, Canada; 6 Current address: Dr. Waleed Al-Katib, Faculty of Health Sciences, American University of Madaba, Madaba, Jordan

## Abstract

**Introduction:**

Osteopontin (OPN) is a malignancy-associated glycoprotein that contributes functionally to tumor aggressiveness. In metastatic breast cancer, we previously demonstrated that elevated OPN in primary tumor and blood was associated with poor prognosis.

**Methods:**

We measured OPN in plasma by ELISA, and in tumors by immunohistochemistry, in 624 (94%) and 462 (69%), respectively, of 667 postmenopausal women with hormone responsive early breast cancer treated by surgery followed by adjuvant treatment with tamoxifen +/− octreotide in a randomized trial (NCIC CTG MA.14; National Cancer Institute of Canada Clinical Trials Group Mammary.14).

**Results:**

Plasma OPN was measured in 2,540 samples; 688 at baseline and 1,852 collected during follow-up. Mean baseline plasma OPN was 46 ng/ml (range 22.6 to 290) which did not differ from normal levels. Mean percentage OPN tumor cell positivity was 33.9 (95% CI: 30.2 to 37.9). There was no correlation between plasma and tumor OPN values. In multivariate analysis, neither was associated with event-free survival (EFS), relapse-free survival (RFS), overall survival (OS), bone RFS or non-bone RFS. An exploratory analysis in patients with recurrence showed higher mean OPN plasma levels 60.7 ng/ml (23.9 to 543) in the recurrence period compared with baseline levels.

**Conclusions:**

The hypothesis that OPN tumor expression would have independent prognostic value in early breast cancer was not supported by multivariate analysis of this study population. Plasma OPN levels in women with hormone responsive early breast cancer in the MA.14 trial were not elevated and there was no evidence for prognostic value of plasma OPN in this defined group of patients. However, our finding of elevated mean OPN plasma level around the time of recurrence warrants further study.

**Trial registration:**

NCT00002864, http://clinicaltrials.gov/show/NCT00002864

## Introduction

Osteopontin (OPN) is a secreted integrin-binding glycophosphoprotein produced in a variety of tissues and cell types, and its association with cancer has been well documented [1-4]. Our studies, and those of others, have demonstrated that OPN contributes functionally to aggressive cell behavior, tumor progression and metastasis. Although OPN is not tumor-specific, its potential as a tumor marker has been described in many malignancies, including breast cancer [2-6].

When measured by immunohistochemistry (IHC), OPN expression in breast cancer tissue has been documented both in tumor cells and infiltrating host cells [7]. In a cohort of 154 patients with lymph node negative breast cancer, we reported that OPN immunopositivity in tumor cells, using a semi-quantitative IHC scoring system, was significantly associated with decreased disease-free and overall survival [7]. Similarly, Rudland’s group reported an association between OPN positivity and poor prognosis in early breast cancer, also using a semi-quantitative IHC scoring system [8-10].

Using an ELISA (enzyme linked immunosorbent assay) that we previously developed and clinically validated [11-13], we demonstrated that compared with levels in healthy women, or patients who had completed treatment for primary breast cancer, plasma OPN levels in women with metastatic breast cancer were elevated and associated with worse survival [11].

In the mid-1990s, we initiated a series of studies in early and metastatic breast cancer to further explore the potential of OPN as a biomarker in tumor tissue and blood. We have already reported [14] the results of one prospective study, which confirmed our earlier results and showed that 99/158 (63%) of women at first presentation with metastatic breast cancer had elevated levels of plasma OPN, and in multivariate analysis elevated OPN was associated with worse overall survival (*P* = 0.03).

Thus, elevated OPN levels in both tumor tissue and blood are well-documented to be associated with poor outcome in the setting of metastatic breast cancer [1-5]. However, much less is known about the prognostic significance of OPN in early breast cancer or when OPN levels rise during breast cancer progression. We thus identified the Mammary (MA).14 phase III clinical trial from the National Cancer Institute of Canada Clinical Trials Group (NCIC CTG) [15], as an opportunity to address these questions. MA.14 provided us with a source of blood and breast tumor tissue samples along with reliable patient data, from women with hormone responsive early breast cancer in the context of a well-controlled clinical trial. We hypothesized that there would be a relationship between baseline tumor and plasma levels of OPN, that both would have prognostic value in early breast cancer, and that breast cancer recurrence would be associated with elevated plasma OPN levels.

## Methods

### NCIC CTG MA.14 clinical trial

In September 1996, the NCIC CTG opened a phase III clinical trial (MA.14) evaluating the survival benefit of a somatostatin analogue, octreotide Long-Acting Release formulation (LAR), added to tamoxifen in postmenopausal women with early stage hormone responsive breast cancer. Collection of serum, at baseline and several time-points thereafter, for assay of multiple metabolic markers of insulin resistance, was an integrated secondary objective. All patients enrolled in the MA.14 trial provided written informed consent before participation, and ethics approval was obtained by all participating centers, as previously detailed in the primary trial report [15]. For our OPN correlative study, a protocol amendment in November 1998 allowed us to add serial plasma collection for OPN, and we also were permitted to access primary tumor paraffin blocks/slides for OPN IHC studies. Postmenopausal women with operable early breast cancer (T1-3, N0, M0) stratified by hormone receptor status were eligible for the MA.14 trial. Tamoxifen 20 mg/day was given for five years in both arms, and octreotide LAR 90 mg intramuscularly (IM) monthly was given for two years in the experimental arm. Modifications in design related to octreotide LAR toxicity are described in the main trial report [15]. Recruitment started in September 1996, and the trial closed in August 2000 with the accrual of 667 women.

Despite excellent preclinical rationale for the anti-tumor actions of somatostatin analogues, small clinical studies showing breast cancer regression and evidence of greater suppression of insulin-like growth factor by octreotide added to tamoxifen, there was no significant benefit for the combination in terms of event-free survival (HR (hazard ratio) 0.93, *P* = 0.62), relapse free survival (HR 0.84, *P* = 0.31) and overall survival (HR 0.97, *P* = 0.86) at a median follow-up of 7.9 years. Five-year event-free survival for all patients was around 78%. There was no evidence of any significant differences in OPN levels by treatment arm, at any time points and, thus, in our OPN analyses we present data combined from both arms of the trial.

### Sample collection

The plasma sample collection was initiated in November 1998. As this was more than two years after study recruitment started (September 1996), reduced numbers of patients and samples were available at some time points. Plasma samples were shipped at ambient temperature to a central lab by overnight courier, where they were promptly frozen at −70°C until being shipped frozen to the London Regional Cancer Program laboratory for ELISA assessment. Prior to initiation of plasma collection for the MA.14 trial, in a pilot study to assess the potential effect of this collection/storage process, we assessed OPN plasma levels from eight blood samples maintained at room temperature for 4 vs. 24, 48 or 72 hours, and found that measured values did not differ with time at room temperature. The total number of plasma samples available for OPN assays was 2,540, with 624 (94%) of 667 patients having plasma collected at some time. The time-points for collection and numbers of available patients and samples are summarized in Table 1. Primary statistical analyses utilized all samples at each time point, but some exploratory analyses examined data from paired samples for recurrent and non-recurrent patients. Prognostic analyses examined baseline IHC and plasma OPN effects on multiple survival outcomes. To maximize inclusion of patients in these investigations and given the low likelihood of tumor recurrence (2 in 388 patients) in the first four months after randomization, we combined pre-randomization and four-month results, giving a “baseline” assessment of OPN in 388 (58%) of 667 patients. When there were multiple assessment results for a patient during the pre-randomization and four-month time-points, they were averaged to yield a baseline OPN plasma value. For measurement of tumor tissue OPN, it was possible to obtain primary tumor blocks/slides for OPN IHC in 462 (69%) of 667 patients accrued to MA.14. In total, 647 (97%) of patients had either measurement of OPN in primary tumor (by IHC), or one or more measurements of plasma OPN (by ELISA).

**Table 1:** Available samples

**Total accrual**		**667 patients**
Total patients with samples for OPN analyses		
Plasma	- baseline/4 m	388 (58%)
	- any time	624 (94%)
Tumor tissue for IHC		462 (69%)
Total number plasma samples		2,540
Timing - plasma samples/number of patients		
Baseline (pre-randomization)		314/301
4 m		374/361
8 m		396/391
12 m		406/401
16 m*		33/29
24 m		540/487
36 m		206/159
48 m*		29/29
60 m		220/211
Recurrence		22/20

*later protocol amendment eliminated these sample collections. IHC, immunohistochemistry; OPN, osteopontin.

For comparison with MA.14 study samples, 90 plasma samples from healthy women were collected under a protocol approved by the University of Western Ontario Research Ethics Board. Volunteer donors were hospital staff who contributed blood samples for the purpose of establishing normal levels of various blood components, including OPN. The only data collected on these individuals were sex and age.

### OPN analytic methods

OPN was measured in plasma samples by ELISA (Human Osteopontin EIA Kit, catalogue #ADI-900-142, Enzo Life Sciences, Ann Arbor, MI, USA) as previously described [16]. This assay uses two monoclonal antibodies whose epitopes have been defined [16]. It should be noted that one of these antibodies recognizes an epitope that is destroyed by thrombin cleavage and, thus, the ELISA can be used with plasma but not with serum. Samples from MA.14 were measured in duplicate and from healthy women in triplicate, and the values for each sample were averaged.

Formalin-fixed, paraffin-embedded primary tumor samples were assessed for OPN expression by IHC using an immunoperoxidase technique, as previously described [7]. The primary antibody used was the monoclonal antibody mAb53 (Enzo Life Sciences, catalogue #ADI-905-629). Biotinylated secondary antibody was from the LSAB2 kit (Dako, Burlington, ON, Canada). Stained slides were evaluated by light microscopy in a blinded fashion by two pathologists (ABT, WAK).

Tumor cell staining for OPN was determined as a continuous variable of the percentage of cells staining (regardless of intensity), as well as using two different semi-quantitative systems. The first was the non-linear system of Allred *et al*. [17], which assigns proportion and intensity scores as follows: proportion as the estimated fraction of tumor cells staining (0 = none; 1 = <1/100; 2 = 1 to 10%; 3 = 10% to 1/3; 4 = 1/3 to 2/3; 5 = >2/3, and intensity as the estimated average staining of tumor cells (0 = none; 1 = weak; 2 = moderate; 3 = strong). The Allred score is then the sum of proportion and intensity scores (0 for negative staining and 2 to 8 for positive staining ranges). The second, H-scoring system of McCarty [18] is a linear system which quantifies the proportion of tumor cells in each intensity category (0 = no staining; 1 = weak; 2 = moderate; 3 = strong). The percentage of weakly stained cells is multiplied by 1, the percentage of moderately stained cells by 2, and the percentage of strongly stained cells by 3; the total of these three categories then gives the H-score (from 0 to a maximum of 300). As OPN staining of tumor-infiltrating inflammatory cells (histiocytes and T-lymphocytes) is generally intense in nature, semi-quantitation of proportion of inflammatory cells staining was performed using a 0 to 3+ system, such that 0 = no staining of tumor infiltrating inflammatory cells; 1+ = scattered OPN positive tumor infiltrating inflammatory cells, 2+ = moderate numbers of positive tumor infiltrating inflammatory cells; and 3+ = numerous positive tumor infiltrating inflammatory cells.

### Definition of MA.14 endpoints

The primary end point of MA.14 was event-free survival (EFS); secondary end points were relapse-free survival (RFS) and overall survival (OS) [15]. As in a serum beta C-terminal telopeptide study with MA.14 samples [19], we also considered here bone and non-bone RFS. Censoring was at the longest follow-up.

### Statistical analysis

Our study utilized the MA.14 final analysis database, median follow-up 7.9 years. Baseline patient and primary tumor characteristics are provided in Table 2. There were no significant imbalances (*P* = 0.17 to *P* = 1.00) in treatment arm or stratification factors for patients included in i) tumor IHC OPN, ii) baseline plasma OPN, iii) primary tumor IHC categorizations of recurrent or non-recurrent and iv) plasma categorizations of recurrent or non-recurrent patients. Formal comparisons, to ensure that patients assessed for OPN were representative of the full population by treatment assignment and trial stratification factors of lymph node status, receipt of adjuvant chemotherapy and hormone receptor positivity, utilized exact Fisher tests.

**Table 2:** MA.14 baseline patient and tumor characteristics

	**Total MA.14 trial**		**IHC OPN cohort**		**Plasma OPN cohort**	
	**Number**	**%**	**Number**	**%**	**Number**	**%**
**Total**	**667**	**100**	**462**	**100**	**388**	**100**
**Age at allocation (yrs)**						
<60	**329**	**49**	**220**	**48**	**193**	**50**
≥60	**338**	**51**	**242**	**52**	**195**	**50**
**Race**						
Caucasian	**644**	**97**	**445**	**96**	**375**	**97**
Not Caucasian	**23**	**3**	**17**	**4**	**13**	**3**
**Performance status (ECOG)**						
0, unknown	**520**	**78**	**362**	**78**	**305**	**79**
1, 2	**147**	**22**	**100**	**22**	**83**	**21**
**T pathologic classification**						
1, *in situ*	**389**	**58**	**269**	**58**	**240**	**62**
2, 3A, 4, unknown	**278**	**42**	**193**	**42**	**148**	**38**
**N pathologic classification**						
0	**352**	**53**	**243**	**53**	**206**	**53**
1, 2, unknown	**315**	**47**	**219**	**47**	**182**	**47**
**Breast surgery type**						
Total mastectomy	**260**	**39**	**174**	**38**	**146**	**38**
Other, segmental	**407**	**61**	**288**	**62**	**242**	**62**
**Number of positive nodes**						
0	**352**	**53**	**243**	**53**	**206**	**53**
1 to 3, 4+, unknown	**315**	**47**	**219**	**47**	**182**	**47**
**ER/PR status**						
Negative, unknown	**62**	**9**	**33**	**7**	**33**	**9**
Positive	**605**	**91**	**429**	**93**	**355**	**91**
**Adjuvant chemotherapy**						
None	**445**	**67**	**312**	**68**	**236**	**61**
Concurrent, sequential	**222**	**33**	**150**	**32**	**152**	**39**
Tumor grade*						
Unknown	**NA**		**0**	**0**	**84**	**22**
1	**NA**		**74**	**16**	**48**	**12**
2	**NA**		**232**	**50**	**150**	**39**
3	**NA**		**156**	**34**	**106**	**27**
Histology*						
No special type	**NA**		**446**	**97**	**290**	**75**
Special type (unknown)	**NA**		**16**	**3**	**14 (84)**	**4 (22)**
Lymphovascular invasion*						
Unknown	**NA**		**0**	**0**	**84**	**22**
No	**NA**		**414**	**90**	**274**	**71**
Yes	**NA**		**48**	**10**	**30**	**8**

NOTES: As there were no significant differences in survival outcomes in the main MA.14 trial for Tamoxifen vs Octreotide, the two arms are combined.

*Only evaluated for OPN study.

ECOG, Eastern Cooperative Oncology Group; ER, estrogen receptor; NA, not available; OPN, osteopontin; PR, progesterone receptor.

Box-Cox transformations were considered for both IHC OPN (percentage positive; Allred score; H-score) and plasma OPN for variance stabilization transformation and to improve the assumption of normality during statistical tests; mean values and 95% confidence limits (CI) reported here are back-transformations to laboratory units as follows: 1) IHC OPN percentage cells staining (underwent Box-Cox power 0.5 transformation); 2) IHC OPN H-score (underwent Box-Cox power 0.5 transformation); 3) IHC Allred score 0 to 8 (no transformation); 4) Inflammatory cell OPN, by intensity (0, 1, 2, 3); 5) Plasma OPN (logarithmic transformation). Box plots were also performed on plasma levels in MA.14 for patients at baseline; non-recurrent patients; and recurrent patients at baseline, prior to the recurrence period and in the recurrent period.

Factors considered in exploratory time to event analyses were the MA.14 baseline patient and tumor characteristics [15], IHC and plasma OPN, and newly assessed pathologic factors of tumor grade, histology and lymphovascular invasion. Univariate analyses utilized a stratified log-rank test, with adjustment by MA.14 trial stratification factors, lymph node status, estrogen and progesterone receptor status, and adjuvant therapy [15]; for consistency with our previous study [7] and those of others [8-10], the univariate cut-point for Allred score was 0 to 4 vs >4. Multivariate analyses employed step-wise forward stratified Cox regression, with *P*-values determined by the likelihood ratio criterion, and a factor added if *P* ≤0.05 (χ^2^_(1)_)_._ Factors entered into the multivariate analysis included continuous OPN, treatment arm, patient age, race, weight, body mass index, Eastern Cooperative Oncology Group (ECOG) status, tumor size and grade, lymph node status, surgery type, lymphovascular invasion, IGF-1 and IGFBP-3 and C-peptide. Pearson and Spearman correlation coefficients were used to examine the association of primary IHC OPN and baseline plasma OPN. Individual patient differences in plasma OPN between clinical windows were compared with matched t-tests utilizing transformed data, and then individual patient values were back-transformed to laboratory units for plots connecting patient values for different clinical windows.

Normative plasma OPN values were established with 90 female healthy controls, age range 19 to 59 years, assessed in triplicate and averaged. Population OPN levels were characterized with Box plots for OPN in healthy women.

## Results

Based on our original hypotheses and the inventory of samples, our specific aims were 1) to evaluate whether baseline/four-month plasma OPN levels were prognostic for EFS, RFS, bone or non-bone RFS and OS; 2) to evaluate whether primary tumor OPN IHC levels were prognostic for EFS, RFS, bone or non-bone RFS and OS; 3) to explore whether tumor OPN expression was associated with baseline plasma OPN levels; 4) to explore individual patient changes in plasma OPN from baseline/4 months to recurrence. For aim 4) we utilized clinical windows to group individual patient OPN values: (a) baseline (pre-randomization/4 months); (b) recurrent cases (Period 1: 8 months after randomization to 18 months prior to recurrence; Period 2: <18 months from recurrence until collection stopped), (c) non-recurrent cases (baseline/4 months; any other time).

In the 90 healthy women, mean plasma OPN was 28.4 ng/ml, median 26.3 and range 11.8 to 109. The upper 97.5% cut-point was 95 ng/ml. There was only a weak correlation of OPN with age (Pearson correlation coefficient 0.20, *P* = 0.06; Spearman correlation coefficient 0.26, *P* = 0.02), with a trend for increasing OPN levels with rising age (Additional file 1: Figure S1). The values obtained in this larger sample of healthy women are consistent with results from our previous cohort of 35 pre- and post-menopausal healthy women, median OPN 47 ng/ml), range 22 to 122 [11,12]. Although the median value was higher in the earlier cohort, the upper limits of the range are quite similar.

As outlined in Table 1, plasma OPN was measured in 2,540 plasma samples. In total, 624 (94%) patients had OPN results at some time. The geometric mean for baseline plasma OPN level, in 388 women, was 46 ng/ml (range 22.6 to 290; Additional file 2: Figure S2 (top, bottom), plotted versus IHC OPN, as both percentage cells staining and H-score). The geometric mean for 193 women allocated tamoxifen was 45.3 ng/ml (95% CI: 43.4 to 47.3 ng/ml), and for 195 women allocated tamoxifen with octreotide LAR, 46.8 ng/ml (95% CI: 44.5 to 49.1 ng/ml). Seven women (1.8%) had baseline values above 95 ng/ml, and only three women (0.8%) had baseline values above 122 ng/ml, our previously reported [11,12] upper limit of normal OPN levels (147.7, 265.1, 289.8 ng/ml). The few patients with high baseline plasma OPN were not observed to have any specific stratification factor profile. The mean cut-point for baseline plasma OPN level, in univariate analysis, did not show any association with EFS (*P* = 0.85), RFS (*P* = 0.28), OS (*P* = 0.06), bone RFS (*P* = 0.23) and non-bone RFS (*P* = 0.45), nor was it significant in the multivariate model.

**Figure 1:**
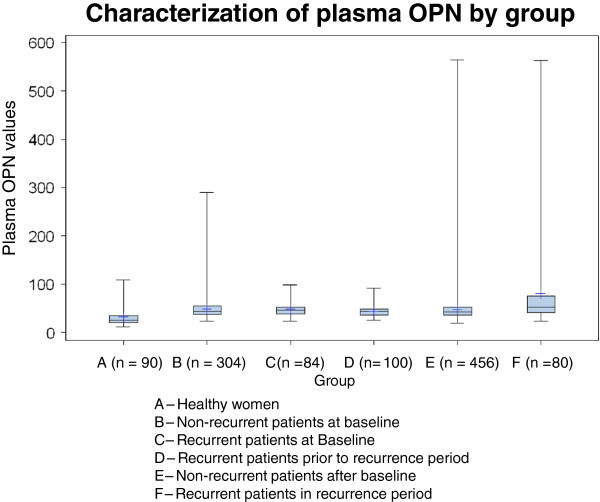
**Characterization of plasma OPN by group.** This figure visually compares plasma OPN levels of healthy women (A = 90 pts) with levels in women who never had recurrence, at baseline (B = 304 pts) and after baseline (E = 456 pts); and with levels in women who had recurrence, at baseline (C = 84 pts), in Period 1 (D = 100 pts) and Period 2 (F = 80 pts). The boxes show OPN values between the 25^th^ and 75^th^ percentiles, with whiskers showing ranges. OPN, osteopontin.

Figure 1 visually compares OPN levels (mean (range) in ng/ml) across six groups, using data from the maximum number of samples available at each time-point. Healthy women (A = 90 women) 28.4 (11.8 to 109) are compared with breast cancer patients in MA.14; those who never had recurrence, at baseline (B = 304 pts) 46.0 (22.6 to 290) and after baseline (E = 456 pts) 43.9 (19.2 to 564); and those who had recurrence, at baseline (C = 84 pts) 46.2 (22.8 to 98.1), in Period 1 (D = 100 pts) 42.8 (25.6 to 92.2) and Period 2 (F = 80 pts) 60.7 (23.9 to 563). In Figure 1, the boxes show OPN values between the 25^th^ and 75^th^ percentiles, with whiskers showing ranges. There is a wider spread of OPN values above the median in samples measured during recurrence (Period 2) in the 80 patients who experienced recurrence (Group F). Three of 490 (0.6%) patients who never had recurrence had OPN levels >122 ng/ml at any time, and 8 of 80 (10%) with recurrence had OPN levels >122 ng/ml, all during the recurrent Period 2. A plot (data not shown) including only patients with paired samples in Groups B, E (270 patients) and C, D, F (31 patients) showed a similar distribution of OPN levels.

**Figure 2:**
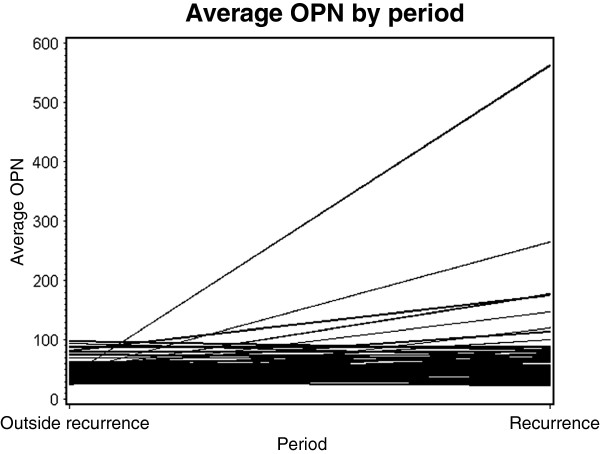
**Average OPN plasma levels in recurrent period vs non-recurrent period for 66 patients.** This figure graphically shows individual patient changes in plasma OPN for 66 patients with recurrence, from the time period prior to recurrence (Period 1) to the recurrence period (Period 2), who had samples available in both time periods. OPN, osteopontin.

Figure 2 graphically shows individual patient changes in plasma OPN for 66 patients with recurrence, from the time period prior to recurrence (Period 1) to the recurrence period (Period 2), who had samples available in both time periods. A matched t-test indicated a significantly higher plasma OPN in the recurrent period (mean 57.3 ng/ml) than at baseline/4 months (mean 46.4 ng/ml) (*P* = 0.002).

When tumor tissue OPN positivity was defined and assessed as the percentage of cells staining by IHC as a continuous variable (regardless of intensity), of the 462 primary tumors assayed, the mean percentage of OPN tumor cell positivity was 33.9 (95% CI: 30.2 to 37.9), and was not significantly different for 105 patients who subsequently developed recurrence (32.4 (95% CI: 24.6 to 41.2)) and the 357 who did not (34.4 (95% CI: 30.2 to 38.8)).

In univariate analyses of tumor tissue OPN, when defined and assessed as the percentage of cells staining by IHC, there was no association between primary tumor OPN expression with cut-point at mean and EFS (*P* = 0.34), RFS (*P* = 0.88), OS (*P* = 0.62), bone RFS (*P* = 0.74) or non-bone RFS (*P* = 0.96), nor was inflammatory cell OPN associated with any endpoint (respectively, *P* = 0.55, 0.26, 0.10, 0.27, 0.75). Similar results were obtained when OPN was assessed by H-score. Using an Allred score cut-point < =4 vs > =5, primary tumor OPN expression was not associated with any survival endpoint (respectively, *P* = 0.24, 0.95, 0.20, 0.79, 0.94). Higher tumor grade (HR 1.90, *P* <0.0001), older age (HR 1.68, *P* = 0.0004), higher pathological tumor stage pT (HR 1.65, *P* = 0.0005) and higher pathological node stage pN (HR 9.02, *P* = 0.0026) categories had significantly worse EFS. These same factors had similar associations with RFS and OS; tumor grade, age and pT were similarly associated with bone RFS; and tumor grade and pN with non-bone RFS.

In the multivariate EFS Cox model, high tumor grade (HR 1.72, *P* <0.0001), age >60 yrs (HR 1.47, *P* = 0.02), higher body mass index (HR 1.55, *P* <0.0001) and increasing nodal involvement (HR 13.21, *P* = 0.001) were significantly associated with worse EFS. Primary IHC tumor expression of OPN (the percentage of cells staining, H-score, Allred score) and inflammatory cell OPN, as well as baseline plasma OPN level, were not significantly associated with EFS in the multivariate model. Additionally, none of the OPN factors were associated with RFS, OS, bone RFS or non-bone RFS.

Neither mean percentage OPN staining by IHC nor mean baseline OPN plasma level differed significantly by T stage (T0-1 vs T2 to 4), tumor grade (1 vs 2 vs 3), nodal status (negative vs positive), hormone receptor status (negative vs positive), age (<60 years vs older), race (Caucasian vs other), or body mass index (in quartiles). Baseline plasma OPN levels were not correlated with primary tumor OPN expression (Pearson coefficient 0.02, *P* = 0.73; Spearman coefficient 0.05, *P* = 0.42).

## Discussion

Our correlative marker study of OPN in the setting of the MA.14 prospective clinical trial was designed in 1998, and the protocol was developed based on data from experimental and clinical studies conducted by our group over several years [7,11,12,20-22]. At that time, OPN as a potential tumor marker in breast cancer fit into the category “+” on the Tumor Marker Utility Grading System (TMUGS) proposed by Hayes *et al*. [23], defined as “sufficient data are available to demonstrate that the marker correlates with the biologic process and/or biologic endpoint related to the use and that the marker results might affect favourable clinical outcome for that use. However, the marker is still considered investigational and should not be used for standard clinical practice…”. In July 1997, we had opened a prospective clinical study evaluating the prognostic value of measuring serial OPN levels in women with metastatic breast cancer [14]. The current study was intended to be complementary, and extend our investigations to early breast cancer taking advantage of the infrastructure of an existing prospective clinical trial that included collection of primary tumor blocks, and serial blood samples. The design of this correlative study meets the criteria for Level II evidence in the system proposed by Hayes *et al*. [23] for “Levels of evidence for grading clinical utility of tumor markers”, which outlined a scale of I to V, with Level I providing the best evidence. In 2005 (and updated in 2012), McShane *et al*. published a paper entitled “Reporting Recommendations for Tumor Marker Prognostic Studies” [24,25], and although this MA.14 correlative study was designed several years earlier, we have endeavored to follow these recommendations in reporting our results.

In our pilot study [7], tumor cell OPN IHC staining above an optimized cut-point using the Allred scoring system was significantly associated with decreased DFS and OS in univariate analysis, but only with decreased OS in multivariate analysis (including patient age, menopausal status, tumor size, grade hormone receptor status and p53 positivity). Similarly, Rudland’s group has reported that OPN positivity in breast tumor tissue (defined as >5% cells staining by IHC), in women with operable Stage I and II breast cancer, was associated with shorter survival [8,9]. In multivariate analysis, our current study shows no significant association between tumor cell OPN expression, scored in several different ways, and any survival outcome. These findings differ from our previous pilot study [7] and those of others [8-10]. The MA.14 trial is larger than previous studies, and recruited a uniform population of postmenopausal women with hormone receptor positive tumors. In previous studies all patients were diagnosed before 1996 (sometimes as far back as 1972) when recruitment started for the MA.14 trial. Better systemic treatments in the modern era, which are associated with steadily improving survival in women with early breast cancer [26], may have reduced the adverse effects of OPN in primary tumors, accounting for the discrepancy between studies. In addition, the patients in this study are all postmenopausal, most are ER or PR positive, and all received tamoxifen. It is possible that OPN may not have the same prognostic significance in this subgroup of patients. In particular, little is known about how tumor cell OPN expression is affected by estrogen antagonists, although it is known that the OPN promoter can respond to both estrogen receptor alpha and estrogen-related receptor [27-29]. If OPN is indeed down-regulated by tamoxifen use, its prognostic impact in this patient population may have been negated by the therapy.

In our first study of plasma OPN in 35 healthy female volunteers, the median level was 47 μg/L (47 ng/ml) with a range 22 to 122 ng/ml, with no significant differences in premenopausal versus postmenopausal women, or according to menstrual status [11,12]. In 44 women who had completed treatment for early breast cancer at least six months earlier, the range of values was similar, 15 to 117 μg/L, but the median level was higher at 60 μg/L [11]. The mean baseline OPN level of 46.0 ng/ml in MA.14 is very similar to the median level we reported for healthy women [11]. We have also shown a similar range of OPN values in 26 healthy male volunteers, median 56 ng/ml, range 26 to 98, using the same ELISA system (Thoms *et al*. [30], Supplemental file, Table 3a in [30]). As all baseline samples for MA.14 were taken within 12 months of primary surgery (for those receiving adjuvant chemotherapy), or within 6 months (no chemotherapy), and this was a population at relatively low risk for recurrence, it is likely that these baseline OPN levels represent a true disease-free (or microscopic residual) state. The 44 women in our pilot study [11] were clinically disease-free after treatment for early breast cancer, but blood for OPN was taken at variable intervals (months to years) after the primary diagnosis. These women were not followed subsequently to determine if and/or when they suffered relapse, with the possibility that some had active subclinical recurrence, which may account for the higher median OPN level (60 ng/ml) compared with healthy volunteers. Thus, in MA.14 it is not necessarily surprising that the mean OPN level of 60.7 ng/ml observed during the recurrence period, for women who developed recurrence, is similar to that found in non-recurrent patients in our pilot study.

In our current study of 90 healthy women, the maximum plasma OPN level was 109 ng/ml, and in healthy male volunteers it was 98 ng/ml [30]. In our first study of healthy female controls, the maximum OPN value was 122 ng/ml [11]. Based on the highest value we have seen in normal controls (122 ng/ml), elevated baseline OPN levels were only seen in 3 of 388 (0.8%) of the women in MA.14. It is interesting that these three patients were in Group B and did not develop recurrence during the follow-up period of the trial. Thus, our results do not suggest that baseline plasma OPN is a useful prognostic marker in this patient population. Our results further did not find an association between levels of OPN in plasma at baseline and tumor tissue in this cohort, perhaps due to the finding that the vast majority of baseline plasma OPN values were not elevated above normal levels.

However, serial assays in MA.14 have provided some intriguing hints about OPN as a marker of recurrence/metastasis. As shown in Figure 1, a higher median and upward spread of OPN levels are seen in Group F patients, those with recurrence during the recurrence period, in comparison to the same patients prior to recurrence (Groups C and D). Several patients in Group F had very high OPN levels (maximum 563 ng/ml). We speculate that high OPN levels during follow-up in some Group E patients, without documented recurrence, could in fact represent recurrence as yet undetected clinically. Elevated plasma levels of OPN are associated with poor prognosis in patients with metastatic breast cancer. The present study suggests that baseline OPN plasma levels in postmenopausal patients with hormone responsive early breast cancer receiving tamoxifen may not have the same prognostic value. However, the elevated plasma OPN levels seen in some patients post-baseline suggests that serially monitoring of OPN levels may be of use as an early indicator of relapse.

Our OPN study has several strengths. There were strong preclinical and some clinical data supporting our interest in exploring OPN as a clinical biomarker in breast cancer in the metastatic setting, but its role in early stage disease was relatively unexplored. The patient population accrued to the MA.14 trial was well defined, and details are given in the final trial report [15]. Multiple patient and tumor prognostic factors were collected, and could be entered in multivariate analyses. Our assays are well validated and reported [7,11,16]. We developed pre-specified hypotheses, and a correlative study plan that was compatible with the trial protocol. The results of our IHC and plasma OPN assays were provided to the NCIC CTG MA.14 study statistician (JWC), who developed the detailed statistical plan and performed the analyses.

Our current study also has several limitations - the relatively short duration of sample collection (five years) at a time when improved treatments for breast cancer may have delayed or even prevented relapse; wide spacing of samples after the first year (every 12 months); and the small numbers of samples (22 in 20 patients) actually obtained at the documented time of relapse. After definitive treatment of primary breast cancer, loco-regional recurrence and/or metastasis will be subclinical for a variable period of time (depending on aggressiveness) before the date of recurrence is documented for trial outcomes. Thus, we defined a recurrence time window (samples collected up to 18 months before recurrence) which expanded the number of available samples for statistical purposes, but may have blunted the comparison of OPN levels between baseline and recurrence. Additionally, the ELISA used here, although clinically validated [7,11,16], may detect only a subset of total blood OPN, for example, not proteolytic fragments of OPN or OPN bound to Factor H [31]. Finally, this trial provides no information about plasma OPN levels before removal of primary breast cancer, as all women had undergone definitive surgery before entering the MA.14 trial and providing their first blood sample. The timing of the baseline samples may also explain the lack of correlation between primary tumor expression of OPN and mean baseline plasma OPN level.

Our hypothesis that there would be a relationship between baseline tissue and plasma levels of OPN was not supported by our data. Given that blood OPN levels can come from many sources (tumor tissue, remodeling vasculature, inflammatory cells and bone [1,2,5]), and vary with the timing of baseline samples post-resection of primary breast cancer, this finding is perhaps not surprising. Similarly, our hypothesis that tissue and plasma levels would have prognostic value in early breast cancer was not supported in multivariate analysis. This difference from previous studies may be due to improved overall prognosis for women with early breast cancer associated with better adjuvant treatments. In addition, or alternatively, the difference may be related to the specifics of the present population (postmenopausal, mainly ER/PR positive, receiving tamoxifen) and the possible estrogen-sensitivity of the OPN promoter. Finally, the elevation of OPN in post-baseline plasma samples, found in both recurrent and non-recurrent patients (some of whom might have recurred at a later date), suggests that there may be a role for serial measurement of plasma OPN to monitor for recurrence, in the setting of early breast cancer. This latter idea is worthy of further study.

## Conclusions

Elevated levels of osteopontin (OPN) protein in plasma or tumors from metastatic breast cancer patients have been associated with poor survival. OPN thus has been proposed as a biomarker of breast cancer progression. However, little was known about its prognostic significance in early breast cancer. Here, we measured OPN in tumor tissue and serial plasma samples from hormone responsive patients with early breast cancer enrolled in the MA.14 trial. In multivariate analysis neither plasma nor tumor OPN provided prognostic information. Thus, tumor or plasma OPN levels may be of limited prognostic value in postmenopausal hormone responsive early breast cancer, although this would need to be confirmed on a broader patient population. However, our finding of elevated plasma OPN in post-baseline samples of a small number of patients suggests that further studies exploring OPN plasma levels around the time of recurrence would be worthwhile.

## Abbreviations

EFS: Event-free survival; ELISA: Enzyme linked immunosorbent assay; HR: Hazard ratio; IHC: Immunohistochemistry; NCIC CTG: National Cancer Institute of Canada Clinical Trials Group; OPN: Osteopontin; OS: Overall survival; RFS: Relapse-free survival; TMUGS: Tumor Marker Utility Grading System.

## Competing interests

AFC received in-kind research support and royalties from Enzo Life Sciences, Inc., for sale of OPN antibody products. The other authors have no competing interests to disclose.

## Authors’ contributions

VHCB, ABT, JWC and AFC designed this correlative study to the MA.14 trial and wrote the initial draft of the manuscript, and all authors contributed to critical revision and editing of the manuscript. PHA conducted the OPN ELISA studies. COP carried out the IHC studies. ABT and WAK read and scored the IHC results, assisted by COP and PHA. VHCB, JWC, LES, KIP and MNP participated in the MA.14 trial and the design of this correlative study associated with this trial. CFW was responsible for coordination of the MA.14 trial and preparation of the database for analysis. JWC and LH conducted the statistical analyses. All authors read and approved the final manuscript.

## Supplementary Material

Additional file 1: Figure S1 Average plasma OPN by age for 90 healthy women. Click here for file

Additional file 2: Figure S2 Plot of baseline plasma OPN in 388 women vs. tissue OPN measured by IHC. Top panel, OPN tissue levels measured as the percentage of cells staining; Bottom panel, OPN tissue levels measured as H-score. Click here for file
