# Supplementary figures and images for: Assessment of osteopontin in early breast cancer: correlative study in a randomised clinical trial
Source: Breast Cancer Res. 2014 Jan 22;16(1):R8. doi: 10.1186/bcr3600 (PMC3978736; doi:10.1186/bcr3600)

## Slide 1
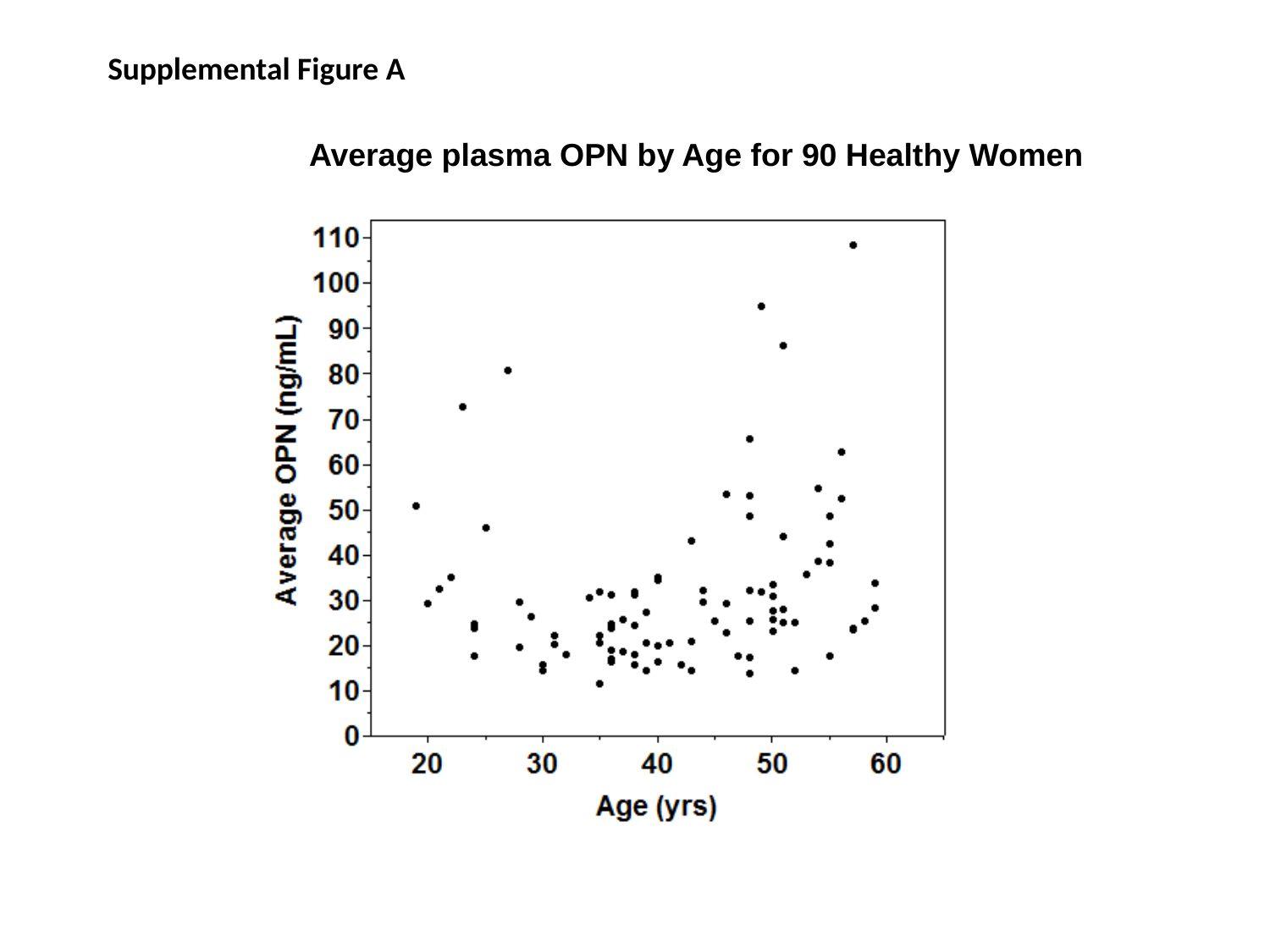

Supplemental Figure A
	Average plasma OPN by Age for 90 Healthy Women

Supplement: Additional file 1: Figure S1 — Average plasma OPN by age for 90 healthy women. [file bcr3600-S1.pptx]

## Slide 1
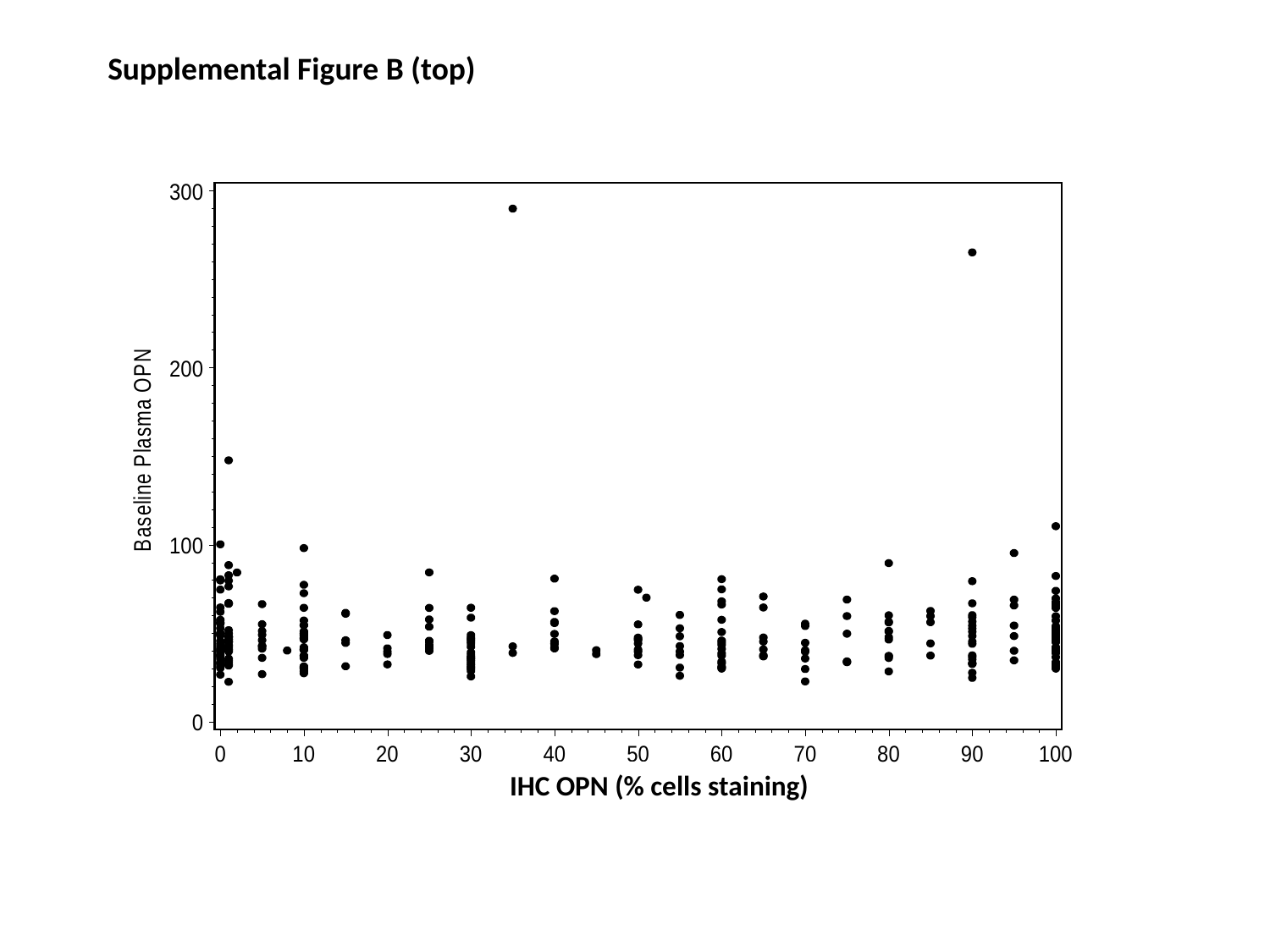

Supplemental Figure B (top)
IHC OPN (% cells staining)

## Slide 2
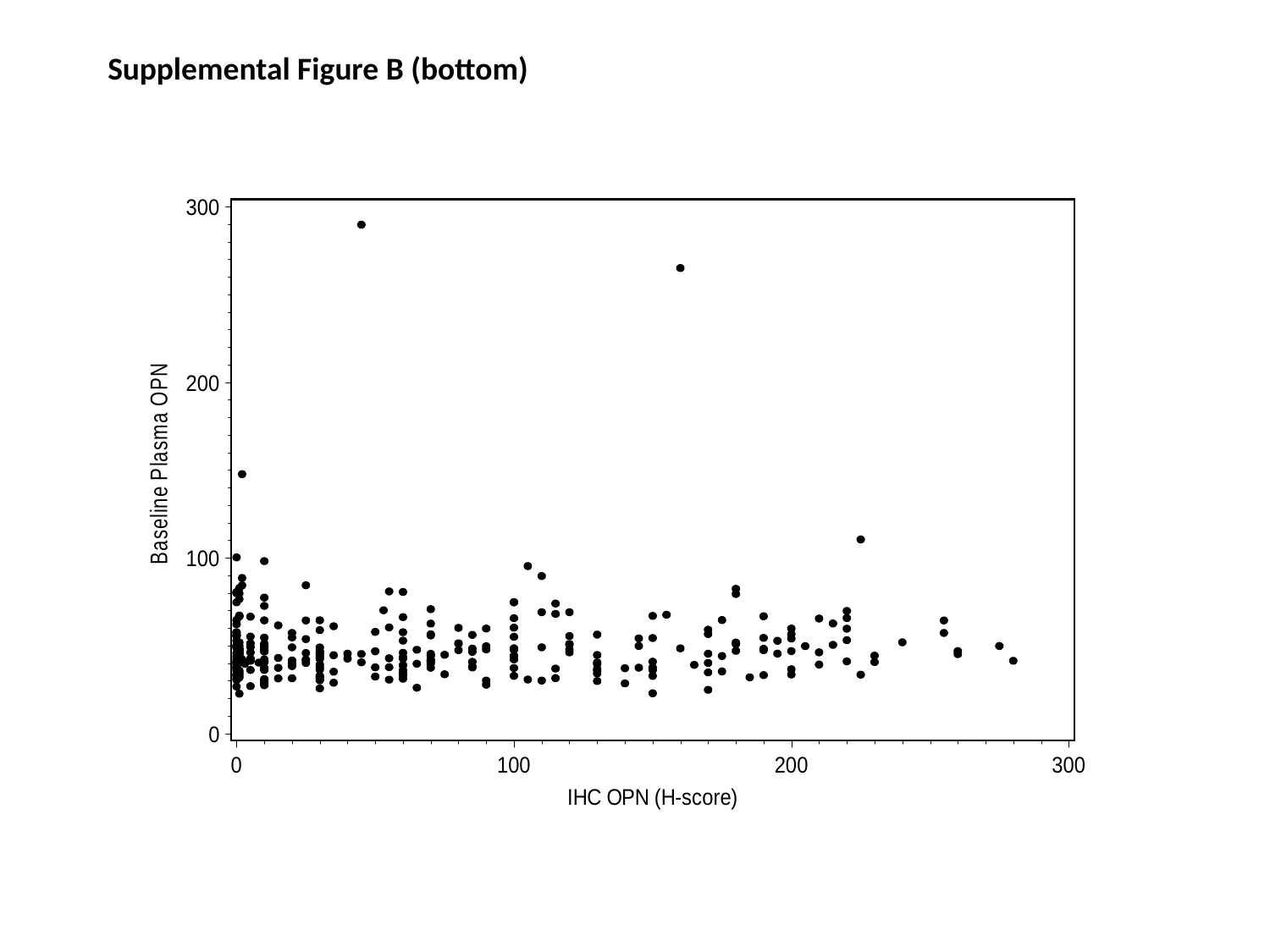

Supplemental Figure B (bottom)

Supplement: Additional file 2: Figure S2 — Plot of baseline plasma OPN in 388 women vs. tissue OPN measured by IHC. Top panel, OPN tissue levels measured as the percentage of cells staining; Bottom panel, OPN tissue levels measured as H-score. [file bcr3600-S2.pptx]
